# Application of personalized medicine to chronic disease: a feasibility assessment

**DOI:** 10.1186/2001-1326-2-16

**Published:** 2013-12-18

**Authors:** Ruslan Dorfman, Zayna Khayat, Tammy Sieminowski, Brian Golden, Renee Lyons

**Affiliations:** 1 Present Address: Geneyouin Inc, Maple, ON, Canada; 2 The Rotman School of Management, The University of Toronto, Toronto, ON, Canada; 3 International Centre for Health Innovation, Richard Ivey School of Business, University of Western Ontario, London, ON, Canada; 4 Bridgepoint Chair in Complex Chronic Disease Research, TD Scientific Director, Bridgepoint Collaboratory for Research and Innovation, Toronto, Ontario, Canada; 5 Dalla Lana School of Public Health, The University of Toronto, Toronto, ON, Canada; 6 Institute of Health Policy, Management and Evaluation, University of Toronto, Toronto, Ontario, Canada; 7 Neurorehabilitation, Bridgepoint Hospital, Toronto, Ontario, Canada

**Keywords:** Stroke, Stroke treatment, TIA, Pharmacogenetics, Statins, Clopidogrel, Warfarin, Antihypertensive, Antidepressants, Patient outcomes

## Abstract

Personalized Medicine has the potential to improve health outcomes and reduce the cost of care; however its adoption has been slow in Canada. Bridgepoint Health is a complex continuous care provider striving to reduce the burden of polypharmacy in chronic patients. The main goal of the study was to explore the feasibility of utilizing personalized medicine in the treatment of chronic complex patients as a preliminary institutional health technology assessment. We analyzed stroke treatment optimization as a clinical indication that could serve as a “proof of concept” for the widespread implementation of pharmacogenetics. The objectives of the study were three-fold:

1. Review current practice in medication administration for stroke treatment at Bridgepoint Health

2. Critically analyze evidence that pharmacogenetic testing could (or could not) enhance drug selection and treatment efficacy for stroke patients;

3. Assess the cost-benefit potential of a pharmacogenetic intervention for stroke.

Review current practice in medication administration for stroke treatment at Bridgepoint Health

Critically analyze evidence that pharmacogenetic testing could (or could not) enhance drug selection and treatment efficacy for stroke patients;

Assess the cost-benefit potential of a pharmacogenetic intervention for stroke.

We conducted a review of stroke treatment practices at Bridgepoint Health, scanned the literature for drug-gene and drug-outcome interactions, and evaluated the potential consequences of pharmacogenetic testing using the ACCE model.

There is a substantial body of evidence suggesting that pharmacogenetic stratification of stroke treatment can improve patient outcomes in the long-term, and provide substantial efficiencies for the healthcare system in the short-term. Specifically, pharmacogenetic stratification of antiplatelet and anticoagulant therapies for stroke patients may have a major impact on the risk of disease recurrence, and thus should be explored further for clinical application. Bridgepoint Health, and other healthcare institutions taking this path, should consider launching pilot projects to assess the practical impact of pharmacogenetics to optimize treatment for chronic continuous care.

## Introduction

Personalized medicine tailors medical treatment to a patient’s personal history, genetic profile and specific biomarkers [1,2]. Pharmacogenetics (PGx) is an application of personalized medicine, referring to the process where patients are stratified and treated based on their genetic profile, which is used to assess expected drug response and the risk of adverse side effects. Traditional medicine typically relies on the broad application of “standard of care” or “one size fits all” treatments to all patients with a given diagnosis, irrespective of their genetic context. The association between one’s gene variants and drug response rates or risks of adverse side effects to specific medications have been reported in many studies; in response, the US Food and Drug Administration (FDA) has updated the labels of nearly 100 drugs with recommendations for genetic testing prior to their use [3].

A personalized medicine approach to drug treatment decision-making offers several potential benefits. The approach may reduce the rate of adverse drug reactions (ADR) [4], which would likely improve treatment adherence. Greater treatment adherence may then prevent disease recurrence or the onset of secondary complications, leading to better clinical and economic outcomes [5]. Additional economic benefits would be gained by limiting the prescription and reimbursement of costly treatments to only those patients who are most likely to respond positively [6]. Despite these potential benefits, the challenge has been translating the many recent discoveries of PGx markers associated with a specific drug response into routine medical practice. In Canada, progress towards including PGx in clinical practice has lagged many peer OECD countries due to a fragmented regulatory environment and lack of reimbursement models [7]. For example the approval and reimbursement of PGx tests is decided separately in each province, leading to long delays for adoption of the technology. In order for strong regulatory guidelines to be created, it is important to consider the feasibility of PGx in clinical settings.

The Bridgepoint Collaboratory for Research and Innovation, the research institute of Bridgepoint Health (BPH), was established to improve the hospital’s understanding of chronic complex diseases (CCD), develop approaches to improving care and integrate advanced research into practice. To investigate the feasibility of using PGx in a clinical setting, we evaluated the potential implications of PGx on stroke treatment at BPH. BPH is what is known as a complex continuing care facility. The majority of patients at BPH are afflicted with multiple chronic illnesses and are often on multiple medications, posing the additional problem of polypharmacy and its associated potential increase in ADRs. Stroke is the third most common condition treated at BPH. The condition has a significant treatment burden due to the need for prolonged rehabilitation and long-term need for multiple medications. Thus the potential impact of introducing the precision of PGx on improving patient outcomes and healthcare costs for this population is significant and worthy of rigorous exploration.

In Canada, more than 50,000 strokes occur every year, making stroke the leading cause of long-term disability. The financial cost of treatment, including direct treatment and patient/caregiver productivity costs, is estimated to average $50,000 per person within the first six months alone. Average costs per patient range from > $17 K for mild transient ischemic attack (TIA) episodes, to > $53 K for ischaemic and > $56 K for haemorrhagic stroke [8]. The annual economic burden of stroke in Canada is estimated to be $2.5 billion, or ~1% of the country’s GDP [9]. All stroke patients require continuous medication following discharge in order to mitigate their constant risk of stroke recurrence [10,11]. An individual who has suffered a stroke has a 20% chance of having another stroke within two years, and 50% of patients have a second episode within 5 years of the initial stroke. Substantial costs for medication are borne not only by an institution (like BPH) during a patient’s hospital stay but, particularly in the long-term, by patients and payers who must finance medications and other therapies that are prescribed upon discharge. Therefore, even a small reduction in stroke recurrence risk or severity has the potential to yield significant economic benefits.

Although the potential value proposition of applying PGx at BPH could is significant, to date no trial has been conducted to survey the clinical validity of PGx stratification in a complex continuing care context. Complex care patients are typically excluded from randomized clinical trials due to their multiple co-morbidities. However, complex care patients are ideal candidates for PGx stratification [12] which can not only improve quality and cost of care in the hospital, but can also benefit outpatients in perpetuity. A major known risk factor for stroke recurrence is inadequate dosing of anticoagulant medication in patients with atrial fibrillation. With adequate anticoagulation therapy at least 9% of strokes could be prevented [13], however the Canadian Stroke Registry Network found that only 10% of atrial fibrillation stroke sufferers were medicated and had a coagulation index within the advised therapeutic range [14]. Additionally, medications used for stroke prevention are also used for patients with other symptoms and diagnoses at BPH - PGx optimization of stroke medication can therefore be of potential value to multiple patient populations.

For this study, we assessed the potential clinical and economic impact of PGx on preventing stroke recurrence. We conducted semi-structured interviews of healthcare providers, reviewed the literature on PGx genes and stroke, and modeled health and economic outcomes. Our assessment confirmed the initial hypothesis that PGx can be used to optimize medication use by chronically ill patients, minimize the risk of patients’ health deterioration, and reduce the associated in-patient and out-patient costs to the Canadian healthcare system, and the broader economy. We suggest that complex continuing care institutions consider performing proof of concept trials to investigate the use of PGx. These studies will help demonstrate the value of PGx for clinical implementation and may inform clinical guideline recommendations.

## Methods

To assess the impact of personalized medicine we addressed three main areas: drugs, genes and consequences. We sought to answer the following questions:

1. Drugs: What is the current standard of care drug treatment for stroke patients? We conducted semi-structured interviews with hospital’s physicians and pharmacists to determine drug treatments used for stroke patients and clinical protocols used for selecting treatment and dosing regimens for prevention of secondary stroke.

2. Genes: Which genes are linked to responses to current drug treatments for stroke?

We reviewed the literature to identify potential genetic associations with current stroke treatment responses. We queried the PubMed database with the following key words: “stroke risk factors”, “stroke treatment”, “stroke AND pharmacogenetics”, “stroke AND gene”, “warfarin AND gene”, “clopidogrel AND gene”, “statin AND gene”, “stroke AND cost effectiveness”. Peer-reviewed publications spanning 2005 - Feb 2012 were reviewed.

3. Consequences: What are the potential health and economic outcomes of applying PGx to current stroke treatment protocols? The Office of Public Health Genomics of the Centers for Disease Control in the U.S. has developed the ACCE Model [15,16] for evaluation of clinical utility, analytic utility, and clinical validity of genetic diagnostic tests for various diseases [17]. We applied this comprehensive framework to critically evaluate PGx tests used for the selection and dosing of treatments for stroke patients undergoing rehabilitation. The pharmacogenetic tests that were evaluated were screened for genes identified in (2) above. Notably, in this overview we do not provide technical details on specific risk alleles for each gene; only replicated and validated pharmacogenetic markers were considered. Cost efficiency and sensitivity analyses were coded and modeled in Excel. Cost efficiency analyses included data from the published literature and cost of drugs according to the Ontario Drug Benefit Plan formulary. The prices for new medications that have not been added to the Ontario formulary were extrapolated from price of the drug in US market. The sensitivity analysis explored factors determining the cost-efficiency of pharmacogenetic stratification.

## Research findings

### Four main classes of pharmacological treatments are prescribed at BPH for secondary prophylaxis in stroke patients

Current clinical practices for stroke treatment at BPH reflect current Canadian Best Practice Recommendations for Stroke Care [18]. These interventions mitigate the key risk factors such as atrial fibrillation, diabetes, hypertension, and hyperlipidemia. Stroke patients receive medications from the following four classes for continuous stroke prevention [19-25]:

1. Antiplatelet control: aspirin or clopidogrel.

2. Continuous anticoagulant treatment: primarily warfarin for patients with atrial fibrillation (10-15% of all stroke patients), or heparins for patients with deep vein thrombosis prophylaxis (administered to the majority of stroke patients during hospitalization).

3. Hypertension control: angiotensin-converting enzyme (ACE) [26] inhibitors or angiotensin receptor blockers are administered to the majority of patients due to additional benefits of these medications.

4. Statins: prescribed to the majority of stroke patients primarily as anti-inflammatory agents and for the control of hyperlipidemia, [27-30].

### Key pharmacogenetic genes implicated in the efficacy of drugs used to treat stroke

Each of the four classes of drugs used for stroke treatment were analyzed in depth to evaluate the potential impact of PGx on their optimal use:

## Antiplatelet analysis

Antiplatelet medications, which block platelet activation and reduce blood clots are used in virtually all patients at risk of cardiovascular disease. The vast majority of BPH stroke patients are on a single antiplatelet medication, predominantly acetylsalicylic acid (ASA). Table 1 summarizes potential pharmacogenetic genes, i.e., genes variants that may affect how a patient responds to antiplatelet treatments.

**Table 1:** Antiplatelet medications and potential pharmacogenetic genes

**Drug name**	**Brand name**	**Potential pharmacogenetic genes**	**PubMed ID**	**Details**
Acetylsalicylic acid	Aspirin	NTRK1; LTC4S; ITGB3	21390260;	Risk of ADR and lower response-associated alleles
			19862937;	
			12816731;	
			19390185	
Clopidogrel	Plavix	CYP2C19; PON1; P2RY12; ABCB1;	22153531;	Risk or phenotype-associated alleles. Any combination of two of CYP2C19 loss-of-function alleles is associated with diminished platelet response and worse cardiovascular outcomes
			22088980;	
			22028352;	
			21881565;	
			21716271;	
			21497813;	
			21332306;	
			21170047;	
			20801498;	
			20126830;	
			19637098	
Prasugrel*	Effient	None		Better suited for patients who cannot metabolize clopidogrel
Dipyridamoles-ASA	Aggrenox			

^*^Prasugrel is not on the Ontario or British Columbia public formularies, therefore economics were analyzed based on US pricing.

### Aspirin

Approximately 50% of patients at BPH are treated with Aspirin [31] at varying doses (80-325 mg). ASA is regarded as ineffective for those patients who still suffer a stroke while medicated with ASA. As an alternative, those patients are typically prescribed the antiplatelet agent clopidogrel for thrombosis prevention.

### Clopidogrel

Clopidogrel (Plavix®) is a pro-drug that requires activation by the cytochrome P450 CYP2C19 enzyme. Depending on ethnicity, up to 25% of patients have mutations in the *CYP2C19* gene that result in a less active or inactive 2C19 enzyme, potentially leading to reduced drug response. In March 2010, the FDA issued a “Black Box” warning for clopidogrel, citing that about 14% of patients are poor metabolizers of the drug due to *CYP2C19* gene variants. Patients with decreased drug efficacy have a 1.5-3 times greater risk of stroke or death compared to patients who metabolize the drug. This lead the FDA to recommend genetic testing to identify patients who are poor metabolizers [32]. Additional genetic variants which affect clopidogrel metabolism have recently been identified. Variations in the *ABCB1* gene may impact the drug’s efficacy by affecting its absorption [33]. Common variants within the *PON1* gene (Q192R) affect the drug’s activation with QQ192 homozygous individuals exhibiting a higher risk of stent thrombosis than RR192 homozygous individuals [34]. However this finding was not replicated and thought to be a result of interaction with cigarette smoking [35]. Comprehensive pharmacogenetic tests based on pharmacogenetic genes can identify poor metabolizers who are at increased risk of stroke when taking clopidogrel and would therefore require alternative therapies.

### Prasugrel

The alternative antiplatelet drug prasugrel (Effient®), approved in Canada in 2010, is much more expensive than generic clopidogrel and was found to be associated with a higher risk of mortality from bleeding. However, the drug is not affected by genetic variations in the *CYP2C19* gene, and produces a consistent response in patient populations. It is unclear whether clopidogrel or prasugrel is more effective in preventing stroke recurrence and heart attacks. Recent studies indicate that prasugrel is more effective than clopidogrel, however these studies did not account for poor clopidogrel metabolizers in the patient population [36-41]. Pharmacogenetic tests would ensure that patients receive the treatments they are most likely to respond to and could limit the unnecessary use of prasugrel, thereby sparing patients the associated bleeding risk unless the drug is deemed necessary (ie, for non-clopidogrel responsers). Using pharmacogenetics to tailor treatment decision for patients who must chose between these two drug options would reduce the risk of recurrent stroke through improved anticoagulation control. The international Clinical Consortium for Pharmacogenetic Implementation has released recommendations advising pharmacogenetic stratification when considering these drugs [42].

### Cost-effectiveness of applying PGx to clopidogrel

The cumulative average health system cost of ischemic stroke treatment for the first six months in Ontario exceeds $53 K [8] while the cost distribution is substantially higher for severe stroke patients. The cost for stroke patients with a Modified Rankin Score of 4, approaches $100 K and doubles for patients with a Score of 5. Stroke recurrence, when preventative treatment fails due to the patient being a poor metabolizer of the prescribed drug or inadequate dosing of antiplatelet medications, has a major impact on the healthcare system. A recent cost-benefit analysis compared *CYP2C19* genotype-guided antiplatelet therapy to treatment with clopidogrel or prasugrel regardless of patient genotype [43]. The results indicated that genotype-guided antiplatelet therapy was more effective and less costly compared to treating all patients, regardless of genotype, with clopidogrel (ICER -$6760 [95% (CI) -$6720 to -$6790]) or prasugrel (ICER -$11,710 [95% confidence interval (CI) -$11,480 to -$11,950]) [43]. A similar cost/benefit profile from applying pharmacogenetics to clopidogrel/prasugrel treatment was demonstrated in a study of patients with acute coronary syndrome undergoing planned percutaneous coronary intervention [44].

Our cost-effective and sensitivity analyses were aimed at identifying the major factors that determine the efficiency of PGx stratification (Table 2). The model is based on the current distribution of antiplatelet medications being prescribed to BPH patients: 50% of stroke patients are currently treated with ASA, 10% are put on Dipyridamole/ASA (Aggrenox®) and the remaining 40% receive clopidogrel. We compared three alternative scenarios: current treatment protocol vs pharmacogenetic stratification vs random. We assumed that only 80% of the clopidogrel users metabolize the drug and the 20% non-metabolizers are at increased risk of stroke recurrence due to ineffective anticoagulation treatment. We also assumed that application of a genetic test would identify the majority of these non-metabolizers, who would then be prescribed prasugrel. Switching clopidogrel non-responders to prasugrel would reduce the overall stroke recurrence risk for the entire stroke population from 8.2% to 7.4%. The cost of PGx genetic testing of $600 per patient in the clopidogrel subgroup (averaged cost $250) would be offset by avoiding the additional treatment cost of a secondary stroke.

**Table 2:** Cost comparisons of current standard of care for stroke patients at BPH compared with pharmacogenetic stratification (PGx) or a randomized alternative

**Proportion of stroke patients treated with particular medication**	**Average annual cost $**	**Base case (standard of care)**	**PGx stratified**	**Alternative random**
ASA	27	0.5	0.5	0.5
Plavix	941	0.4	0.32	0.2
Aggrenox	601	0.1	0.1	0.1
Effient (estimated cost)	1110	0	0.08	0.2
Proportion of untreated		0.08	0.016	0.04
Recurrence risk untreated		0.2	0.2	0.2
Recurrence risk treated		0.072	0.072	0.072
Cost of treatment failure		53,576	53,576	53,576
Probability of failure		0.082	0.074	0.077
Risk adjusted failure cost^*^		4406	3967	4132
Incremental annual cost of treatment		450	463	484
Cost of PGx test		0	250	0
Total costs Year 1		4856	4680	4615
Discounted costs Year 2^**^		4714	4301	4481
Discounted costs Year 3^**^		4577	4176	4350
Discounted costs Year 4^**^		4444	4055	4224
Discounted costs Year 5^**^		4314	3936	4101
**Cumulative costs:**		**22905**	**21149**	**21771**
**Differential savings**		0	**1756**	**1134**

*Average cost of ischemic stroke treatment in Ontario according to Goeree et al, 2005.

Please note that the costs are substantially higher for more severe stroke patients: costs for patients with Modified Rankin Score 4 = 100,000$CDN and score 5 = 200,000$CDN.

**NPV calculation assumes 3% annual interest rate.

To assess the factors affecting the robustness and sensitivity of this model we modified several variables. The direct cost of PGx testing is not a significant factor and nullified the positive effect of clopidogrel stratification only if the cost exceeded $5,100. Surprisingly the PGx test sensitivity, i.e. the ability to correctly identify poor clopidogrel metabolizers turned out to be the most sensitive parameter in the model. The PGx approach ceases to be cost effective when more than 21% of patients continue on clopidogrel despite being poor metabolizers. Therefore, healthcare providers and policy makers must consider PGx test sensitivity in their patient population in order to achieve the maximum clinical and economic benefits of pharmacogenetic stratification, even at higher costs of genetic testing.

### Summary of pharmacogenetics applied to antiplatelet medications

Conducting a pharmacogenetic-based patient stratification prior to prescribing clopidogrel has the potential to improve treatment efficacy and yield substantial long-term economic benefits for stroke patients and health care funders. As of 2012, clopidogrel has been generalized, resulting in lower costs compared to prasugrel and subsequent savings for the healthcare system on a per pill basis. If clopidogrel dosage is adjusted according to patients’ metabolic profiles, this may result in further savings (not modeled as part of this study).

## Anticoagulation analysis

Warfarin is used broadly in patients with atrial fibrillation, heart valve replacement, recent heart attack, and for venous thromboembolism prophylaxis in patients who have undergone hip or knee replacements. However, warfarin has a narrow therapeutic range: at low doses it does not decrease the risk of stroke, while at higher doses it significantly increases the risk of intracranial bleeding [22,45]. Proper warfarin dosing is challenging due to variation in *VKORC1* and *CYP2C9* genes (Table 3), which impact the clinical efficacy of the medication [46-49].

**Table 3:** Anticoagulant treatments and genes with variants that potentially affect drug response

**Drug name**	**Brand name**	**Pharmacogenetic genes**	**PubMed ID reference**	**Details**
Warfarin	Coumadin	CYP2C9; VKORC1; CALU; CACNA1C; CYP4F2	20200517;	Risk or phenotype-associated alleles. Weekly warfarin dose requirements were lower in those with CYP2C9 loss-of-function alleles as compared with the wild type CYP2C9 genotype
			19794411;	
			20072124;	
			17341693;	
			18250228;	
			20203262;	
			18535201;	
			19297519;	
			19300499;	
			18535201	
Dabigatran*	**Pradax**			
Heparin				
Enoxaparin	Lovenox			

*Dabigatran is not on Ontario or BC formularies, but is used in Canada.

A pharmacogenetic approach for accurate warfarin dosing has been developed based on known variations in *VKORC1* and *CYP2C9* genes [50]. PGx testing can explain ~ 50% of the dose variance in Caucasians and can reduce the time necessary to determine optimal INR (International Normalized Ratio of prothrombin time) in the first few days of drug initiation. Although this reduces incidence of out-of-range INRs, there is little benefit for PGx testing beyond the first two weeks of treatment initiation for patients with empirically established dosage.

Some pharmacogenetic tests are less effective in Asians and African Americans due to inability to capture additional pharmacogenetic variants in these populations. The use of ethnically-optimized biomarkers can further improve PGx-based dose prediction algorithms in these populations [51], allowing continuous and safe utilization effective warfarin treatment. Clinical Pharmacogenetics Implementation Consortium’s guidelines for warfarin dosing have been published supporting the application of genetic testing prior to drug initiation [52].

Prospective clinical trials have investigated the use of a pharmacogenetic approach for warfarin dosage in cohorts of orthopedic patients [53] and patients with atrial fibrillation [54]. The studies demonstrated the superiority of the PGx approach over the current standard of care in reducing the number of adverse effects through genetically guided warfarin dosing within the first days of hospitalization. Additional pharmacogenetic trials of warfarin dosing are currently underway (NCT00927862).

### Alternative anticoagulant therapeutics

**Dabigatran etexilate** (PRADAX™), which was recently approved by Health Canada, has similar efficacy to warfarin but is considered a safer alternative [55], as the drug is not currently known to have pharmacogenetic interactions. However, dabigatran was associated with an increased incidence of gastrointestinal adverse reactions, (35% vs. 24% for warfarin) and no competitive inhibitors for dabigatran are available.

### Cost-effectiveness of warfarin and dabigatran

Dabigatran is ten times more expensive than warfarin [56,57], although the higher cost could be offset by a reduction in secondary stroke risks and associated treatment cost, and absence of INR monitoring, which is required for warfarin users. However, as these cost evaluations did not compare dabigatran against warfarin pharmacogenetic protocols [58] it is still unclear whether dabigatran is indeed more cost-effective than warfarin [56].

An extensive cost-effectiveness analysis conducted by Lutter et al. [59] estimated that genetic testing for warfarin therapy for an individual patient can reduce healthcare costs by $550 ($900 savings minus $350 for the cost of genetic testing). For the U.S. healthcare system, net savings from warfarin PGx could be about $1.1 billion annually while even with BPH’s smaller patient population scale, the long-term savings to the healthcare system could be substantial [24].

### Summary: pharmacogenetics applied to anticoagulants

Although PGx-guided selection of warfarin dosage could be more cost-effective than alternative treatment with dabigatran, the impact of warfarin pharmacogenetics is difficult to assess due to highly variable effects caused by non-genetic factors such as diet. However, if stroke patients undergo genetic testing for clopidogrel-metabolizing genes, the additional cost of genotyping *VKORC1* and *CYP2C9* genes would be very low.

## Antihypertensives analysis

The clinical efficacy of the remaining classes of stroke treatment medications such as angiotensin-converting enzyme inhibitors, angiotensin II receptor (AGTR2) antagonists, and statins are also affected by genetic variation in metabolic genes, as well as in genes encoding the drug’s main targets and proteins involved in the signaling cascades (Table 3). Most stroke patients are prescribed ACE inhibitors for blood pressure control and the anti-inflammatory effects that reduce the risk of stroke recurrence [21,60]. ACE inhibitors have known ADRs, including hypokalemia and drug-induced dry cough and can be substituted with AGTR2 antagonists. Occasionally, administration of ACE or AGTR2 blockers even at the maximal therapeutic doses is insufficient to control hypertension. In such cases, additional medication is prescribed alongside a high dose of ACE or angiotensin II receptor blockers. Simultaneous administration of several antihypertensive compounds the polypharmacy effect, thus contributing to the cumulative risk of ADRs.

### Pharmacogenetics of antihypertensive medications

Although different ACE inhibitors, as well as AGTR2 antagonists, have different pharmacogenetic profiles (Table 4), physicians and especially pharmacists generally do not differentiate between antihypertensive medications within the same therapeutic class –i.e. an ACE inhibitor could be switched for a ACE blocker by a pharmacist, depending on availability, without considering potential differences in pharmacogenetic profiles, as these medications are considered to be therapeutically interchangeable. Pharmacogenetic profiling for antihypertensive drugs is not very cost-effective because treatment optimization through adjustment of drug and/or dose and blood pressure monitoring is easy and inexpensive.

**Table 4:** Antihypertensives and statins: genes with variants that potentially affect the drug response rate or the risk of adverse side effects

**Drug name**	**Brand name**	**Pharmacogenetic genes**	**PubMed ID**	**Effect**
***ACE inhibitors***				
Captopril	Capoten	AGTR1	18347611	Specific variants (s.v.) associated with improved outcomes
Enalapril	Vasotec; Renitec			
Ramipril	Altace; Tritace;			
Perindopril	Coversyl; Aceon	AGTR1; BDKRB1	20712529; 20712529	S.v. associated with improved outcomes
Lisinopril	Listril; Lopril; Novatec;	AGTR1; CLCN6; NPPA	18347611; 18212314	S.v. associated with improved outcomes
***AGTR2 antagonists***				
Losartan	Cozaar; Hyzaar; Lacidipine; Lortaan	CYP2C9	11823761; 11408373	CYP2C9*3 allele is associated with a reduced rate of drug metabolism
Irbesartan	Avalide; Avapro; Irbesarran;	APOB; LDLR	15453913; 15453913	S.v. associated with improved outcomes
Candesartan	Amias; Atacand; Blopress;	KNG1	19584173	S.v. associated with improved outcomes
Valsartan	Diovan			
Telmisartan	Micardis; Pritor			
***Statins***				
Atorvastatin	Lipitor; Torvast	CYP3A4; ABCB1; HTR7;HTR3B; GNB3; USP5; SLCO1B1; ABCC1	17600820; 18851956; 19833260; 18851956	Risk or phenotype-associated alleles
Rosuvastatin	Crestor	CYP2C8; CYP2C19	20178046	
Simvastatin	Zocor; Lipex	CYP3A4; HCR; SLCO1B1; AGTR1; KIF6; HTR7; HTR3B; GNB3; USP5; ITGB3;	11250978; 20403997; 18073581; 18551043; 18222353; 18222354; 18222355; 17600820; 11545752	Risk or phenotype-associated alleles

## Analysis of statins

Some statins, especially simvastatin, are known to cause severe muscle or bone pain, and in some patients can result in severe muscle waiting and kidney failure. Statins exhibit differential pharmacogenetic profiles, and the risk of muscle damage can be alleviated by switching to a different brand. Like antihypertensive drugs, the efficacy of statins can be monitored by testing blood cholesterol and adverse side effects are frequently eliminated by substitution to another brand with a different pharmacogenetic profile. Pharmacogenetic genes implicated in patients’ responses to statins include *SLCO1B1* and *ABCC1*. Both SLCO1B1 and ABCC1 transporters mediate drug uptake from the gastrointestinal tract and genetic variants alter drug uptake, resulting in differing drug responses. For instance, *SLCO1B1* variants block the uptake of flavonoids present in grapefruit juice and statins and missense variants were shown to be associated with simvastatin-induced myopathy [61,62]. Pharmacogenetic testing for *SLCO1B1* variants can significantly reduce the incidence of simvastatin-induced myopathy and allow for wider and safer use of the drug [63], which is the cheapest among statins. However, use of PGx for the selection of statin treatment is not expected to have a major impact on the efficiency of stroke prevention. Therefore, this test could be a useful add-on to other genes in the genetic testing panel, but should not be pursued as a stand-alone test.

### Summary: pharmacogenetics of antihypertensives and statins

PGx may reduce the rate of undesired side effects of antihypertensive drugs and statins, resulting in better treatment adherence after discharge. However, the clinical efficacy of statins, ACE and AGTR2 blockers is more markedly affected by changes in cholesterol levels and blood pressure, respectively. In this case, PGx has a limited impact on drug selection and stroke recurrence risk and recovery. PGx testing for these drugs is typically included in a more extensive screening panel, as the cost of including these variants in a genetic test is negligible.

### Added value of expanded pharmacogenetic testing

At BPH, antidepressants are frequently prescribed to help both stroke and general practice patients cope with debilitating chronic diseases. Like clopidogrel and warfarin, antidepressants are metabolized by the CYP family of proteins and response to some antidepressants, as well as risk of adverse side effects is linked to CYP2C19 and CYP2D6 metabolic activity. Citalopram, the most commonly used antidepressant at BPH, is primarily metabolized by both the cytochrome enzymes, and patients who have gain-of-function mutations in these genes are fast metabolizers and exhibit a poor response to citalopram. Genetic testing for variants in the *CYP2D6* and *CYP2C19* genes can be accomplished using existing CYP panels. A broad CYP450 genotyping or ADME screening panel could potentially improve selection of antidepressant treatments.

### Summary of results

The PGx-guided treatment approach has the potential to optimize drug treatment for complex continuing care patients who take a battery of medications for a prolonged period of time. We have shown that genetic testing for antiplatelet medications and anticoagulants would greatly benefit prevention of secondary stroke, particularly in patients on clopidogrel or warfarin. Specifically, PGx testing for clopidogrel metabolism is strongly recommended by the FDA and leading pharmacogenetic experts. It would be prudent of BPH to consider practical implementation of genetic testing for current and future clopidogrel users. Furthermore, an integrated approach for critical re-evaluation of the entire drug portfolio can add further value at minimal extra cost when expanding basic testing to include other gene variants.

## Discussion

A pharmacogenetic treatment stratification could be critical to improving treatment efficacy and cost-efficiency by optimizing drug selection and dosing according to a patient’s genetic profile. This method is an evidence-based approach and relies on scientifically developed correlations between responses to medications and specific genetic variants. Once the methodology for treatment optimization is established for a particular disease, the same model can be applied for the evaluation and reassessment of other chronic complex diseases.

Polypharmacy is a major issue in CCD treatment, as patients typically receive multiple medications concurrently. In Canada, 1.3 million seniors take five or more medications, and in complex care 80% of patients are concurrently treated with three or more medications [64]. High pill burden leads to poor treatment adherence and contributes to an increased risk of ADRs. Additionally, polypharmacy is a known morbidity and mortality risk factor. Up to 12% of seniors taking multiple medications visit the hospital for an ADR each year. The direct medical costs of ADRs exceed $100 billion annually in the U.S. alone. Implementation of pharmacogenetics in CCD treatment may help to reduce the polypharmacy burden [65,66], and provide specific recommendations to physicians that may help to reduce the error rate in drug prescriptions.

The current study shows that PGx can improve selection and dosing for at least two of the four classes of drugs commonly prescribed to stroke patients at BPH, particularly clopidogrel and warfarin, since these medications can substantially reduce the risk of stroke recurrence. PGx testing for ACE inhibitors and statins is less critical to risk of stroke recurrence, although it could reduce the burden of polypharmacy. PGx-driven treatment optimization is feasible and might have substantial value for multiple conditions as many medications are being used for separate diseases that share the same comorbidities. However, in many instances there is still ongoing debate about the strength of evidence of PGx indications and no clear cost-effectiveness analysis that includes simultaneous modeling of multiple medications and comorbidies which are relevant for complex care. It is unlikely that clinical trials for PGx-driven complex care will be carried out in the near future. Nevertheless, in order to improve care for its patients, BPH needs to consider practical implementation of pharmacogenetics in routine clinical practice in a step-wise manner.

To further investigate the value of PGx testing, a pilot study could be performed using extended genetic testing with the ADME panel. This would help determine the initial dosage of warfarin and allow better use of antihypertensive and antidepressant medications. The economic benefits of such an approach are expected to be substantial and long-lasting. Additionally, long-term research studies with patient follow-up are needed to ensure patient safety in PGx trials and coordination of care. The accompanying economic analysis for PGx-driven treatment optimization has to take into consideration all medications that may differ from patient to patient, necessitating the development of more comprehensive and flexible models.

In theory, PGx could be used in a multitude of clinical settings and disease conditions, however significant barriers exist to broader implementation including: 1) lack of clear clinical guidelines, 2) cost and reimbursement for genetic tests, 3) rapidly evolving technology that needs to overcome regulatory barriers, 4) the need for integration with electronic patient records and better predictive algorithms and 5) training of physicians in genetics, pharmacogenetics and genetic counselling. From our informal discussions with BDP’s physicians it was clear that the clinical team lacked sufficient pharmacogenetics knowledge and the right tools for supporting decision-making. Also, there is no clear reimbursement system to pay for genetic testing.

The direct costs of genetic testing are only a small fraction of the costs associated with personalized medicine in complex care. While proof of concept trials for PGx-driven optimization could be funded through research grants, under the current hospital funding system all costs for personalized medicine will have to come from the hospital’s operations budget. From a purely economic perspective for BPH and other healthcare institutions, there is no financial incentive to support the implementation of PGx approaches despite the huge potential for improving patient outcomes and the *long-term* costs of care through reduction of polypharmacy and risk of ADRs. In order for PGx approaches to be successfully implemented, future funding guidelines need to take into account the long-term benefits of treatment improvement programs.

### Take away messages

• PGx can improve patient outcomes by reducing the risk of stroke recurrence and severe side effects

• PGx has the potential to directly reduce costs of drug treatments both during patient hospitalization and post-discharge

• PGx can have an immediate and long-term impact on patient outcomes and could substantially reduce both direct and indirect healthcare costs while improving quality of life

• PGx implementation requires a long-term outlook on patient outcomes and necessitates revision of hospital funding initiatives

• Extensive CYP or ADME screening panels that test for a large number of medications provide added value and could be more cost-effective to current standard of care

## Competing interests

Renee Lyons, Tammy Sieminowski and Brian Golden - none; Ruslan Dorfman, is a founder and CEO of Geneyouin which is a direct to consumer provider of genetic testing and consulting services; in the past two years Zayna Khayat was employed as a management consultant at SECOR and KPMG LLP for public and private Canadian healthcare system and received fees over $10,000.

## Authors’ contributions

RD and ZK conducted literature review, interviews and drafted the manuscript; TS conducted literature overview and assisted in manuscript writing; RL and BG supervised the project, provided strategic direction and assistant in manuscript writing. All authors read and approved the final manuscript.
